# Determination of Residual Stresses in 3D-Printed Polymer Parts

**DOI:** 10.3390/polym16142067

**Published:** 2024-07-19

**Authors:** Madina Issametova, Nikita V. Martyushev, Abilkaiyr Zhastalap, Layla B. Sabirova, Uderbayeva Assemgul, Arailym Tursynbayeva, Gazel Abilezova

**Affiliations:** 1 Department of Mechanical Engineering, Institute of Energy and Mechanical Engineering, Satbayev University, Almaty KZ-050000, Kazakhstan; isametova69@mail.ru (M.I.); slb2609@mail.ru (L.B.S.); a.uderbayeva@satbayev.university (U.A.); nurtassovna.iqo@mail.ru (A.T.); abilezova_gazel@mail.ru (G.A.); 2 Information Technology Department, Tomsk Polytechnic University, 634050 Tomsk, Russia

**Keywords:** 3D printing, technological process, process parameters, residual stresses, mechanical characteristics, mathematical model, PLA plastic

## Abstract

This paper presents the results of an investigation of the possibility of the reliable determination of the residual stress–strain state in polymers and composites using a combination of bridge curvature, optical scanning, and finite element methods. A three-factor experiment was conducted to determine the strength of printed PLA plastic products. The effect of the residual stresses on the strength of the printed products was evaluated. By comparing the values of the same strength stresses, a relationship between the nature of the stresses and the strength of the samples was found. A tendency of the negative influence of tensile stresses and the opposite strengthening effect of compressive stresses was obvious, so at the same values of tensile strength, the value of residual stress of 42.9 MPa is lower than that of the fibre compression at the value of 88.9 MPa. The proposed new methods of the residual stress determination allow obtaining a complete picture of the stressed state of the material in the investigated areas of the products. This may be necessary in confirming the calculated models of the residual stress–strain state, clarifying the strength criteria and assessing the quality of the selected technological modes of manufacturing the products.

## 1. Introduction

The range of tasks that can be solved using modern 3D-printing systems is expanding day by day [1]. FDM is attracting increasing attention due to its affordability, ease of maintenance, and the growing variety of available materials such as poly lactic acid (PLA), polypropylene (PP), polyethylene terephthalate glycol (PETG), and acrylonitrile butadiene styrene [1].

More and more often, the possibilities of FDM printing are used by engineers and designers in the machine-building industry with the help of new equipment, and the replacement of metal materials can make solving problems easier during the creation of conceptual designs, as well as the production of finished products. The practice of manufacturing die tooling from ABS-M30 plastic is well known [2]. To investigate the possibility of replacing the metal impeller of a multistage centrifugal pump with a polymer impeller, the authors investigate FGF technology [3]. FGF, a method of direct extrusion of polymer granules, is similar in material application to FDM/FFF technology—layer-by-layer cladding by means of plastic filaments (filament). A model with a double-sided punch in the upper die and its counterpart in the lower die for sheet metal drawing were produced by FDM technology [4].

The FDM process is a multi-parameter technological process where the influence of each production factor leads to the accumulation of residual stresses. In principle, in any material processing, residual stresses can lead to the significant deformation or delamination of the printed parts, which can affect the dimensional accuracy and strength of the responsible parts [5,6,7,8].

The formation of the mechanical properties of polymer products is influenced not only by the material structure but also by the processing method. The properties of printed products are much inferior to those of injection-moulded products despite the fact that 3D technologies allow adjusting a large number of printing parameters [9]. To date, not a few results studying the influence of technological and technological parameters of 3D printing on mechanical properties have been obtained. It has been established that the crystallisation of semi-crystalline polymers strongly depends on temperature and, therefore, to a large extent depends on FDM printing parameters [10,11,12,13,14,15].

Increasing the nozzle speed from 30 mm/s to 60 mm/s can reduce the cooling time between the applied fibres/layers, which negatively affects the crystallinity of the polymer and leads to low crystallisation and reduces the tensile strength of the products [16]. The raster pattern directly affects the strength, weight, printing time, and accumulated stresses, which can lead to crack development and delamination [17,18,19].

A full assessment of the influence of technological parameters not only on the level but also on the nature of the residual stresses occurring during FDM printing will allow the creation of the most rational technological process for the production of quality products. In previous studies, it was found that the residual stresses directly depend on the fill density and printing temperature [20]. Researchers conducted a single-factor calculation experiment with the Digimat-AM program, and the results of the study show that there is an inversely proportional relationship between layer thickness, printing speed, and the level of the residual stresses, while the effect of temperature has a directly proportional effect. The influence of the technological factor raster patterns was studied in [21]. The results of the study show that among all the raster patterns examined, the concentric raster pattern showed the lowest deformation (5.5% reduction) and also the lowest residual stresses (21% reduction). All these previous studies have established the influence of one of the technological factors on the level of the residual stresses, and our task was to establish the simultaneous influence of a complex of factors and to derive a mathematical model describing the dependence of the residual stress level on three factors: the extruder head temperature, layer thickness, and filling.

Various methods have been developed to assess residual stresses in additive manufacturing techniques, including X-ray and neutron diffraction, ultrasonic velocity measurements, magnetoacoustic emission, hole-drilling, tool-point indentation, crack pliability assessment, layer removal, etc. For polymer products, Kasavola et al. [22] studied the residual stress in three-dimensional FDM measurements, using the hole-drilling method. The plate surface deformation was analysed using electron speckle interferometry.

A combined inversion method for the determination of the residual stresses in parts printed by FDM technology was proposed by the authors of [23]. The method combines a theoretical model with in situ measurements of the deformation of the bottom surface of the substrate using FBG sensors.

Safronov et al. [24] investigated deformation and residual stresses in beams (rectangular in cross-section) with the curvature fitting of a deformed beam, and the advantage of this process is that the parts can be analysed in a non-destructive manner. Kantharos et al. [25] studied the effect of different parameters on residual stress during the printing process. They performed in situ stress measurement by embedding fibre Bragg grating and sensors in FDM parts. Some researchers have tried to monitor in situ fibre displacement for changes, defects, and other parameters that affect print quality in different ways. In this case, techniques such as optical coherent gradient sensing [26,27] and acoustic emission [28] were used.

Despite the considerable development of technology and modelling, residual stresses are still poorly understood and difficult to determine, especially in anisotropic materials. It is most characteristic of structures produced by additive technologies or composite materials.

Residual stresses in FDM-printed parts were determined with the hole drilling method [29]; to avoid local amplification of the tenso-metric sensor, an optical method, i.e., ESPI (electronic speckle interferometry), is used to measure the surface displacement due to stress relaxation and hence calculate the residual stresses. A new experimental method [30] is to measure the local strain response in terms of displacements in small increments of crack length by electron speckle interferometry. In [31,32], speckle interferometry analyses of the stress–strain state around the hole at loading stages up to fracture are presented.

In the following, we would like to cite a method that served as a basis for our integrated method. The bridge curvature method, which involves measuring the deflection or curvature of a component caused by the addition or removal of material containing residual stresses, is commonly used to determine the thermal stresses after machining. It can be applied to SLM and FDM components. Since additive manufacturing is based on thermal processing of material, it is known that melting successive layers, e.g., to optimise process parameters (such as laser power, scanning speed and strategy, layer thickness, preheating, etc.), has a significant effect on the residual stresses [33,34,35].

The bridge curvature method in conjunction with the probing-hole method [36] consists of measuring displacement in sheared parts using an optical microscope; the three-dimensional measurement approach allows a better study of the strain distribution on the specimen to determine unambiguously the maximum distortion and the associated orientation.

Studies on the use of scanners to determine residual stresses show that this practice is not widely used despite the lower cost of equipment and availability. So, we would like to mention the work [37], where the simulation analysis of the elasticity of the deformation of the surface of an arbitrary shape composite with foam filler is studied. The simulation analysis includes the study of the deformation at different angles and radii. Comparative analysis shows the effectiveness of the deformation prediction model in this work.

All the listed bridge curvature techniques are based on displacement measurement in the substrate, i.e., at the base of the bridge piers, which is uninformative for the overall picture of RS distribution. In the abovementioned works, the optical scanning method was performed on arbitrarily shaped models, which leads to distortion of the results and affects the conclusions about the regularity of the effect on the residual stresses and on the overall strength in general.

One of the goals of this work is to create a unified method for determining residual stress in polymer-printed parts. In this work, the authors have combined three approaches to determine residual stresses. The bridge curvature method, the scanning method, and the finite element modelling of residual stresses were combined. Also, one of the objectives of the work was to determine the influence of the printing parameters of the FDM process on both the overall strength of the specimens and the level of residual stresses.

## 2. Methods and Materials

### 2.1. Bridge Curvature Method

A new qualitative method is currently used to analyse residual stresses in parts manufactured by FDM technology. The method is based on measuring the curvature of a bridge-shaped specimen [38]. After separating the fabricated specimen from the build platform, the internal residual stresses partially relax and the specimen is curled at a certain angle, which gives information about the residual stresses in the synthesised material. The torsion angle α is a semi-quantitative indicator of the amount of residual stress within the part. The dimensions of the bridge model were verified and validated in [39]. Using a finite element model of the specimen and knowing the torsion angle α, quantitative stress values can be calculated [40]. In this research, we propose the definition of a different parameter to find the magnitude of residual stresses. It is proposed not to measure the torsion angle because the angle α is rather small and the calculation gives large errors. We consider it more appropriate to determine the displacements in coordinates located on the main axes of the ellipse of the deformations of the bridge surface.

The proposed improved method consists of determining residual stresses according to the following algorithm.

1. Printing standard samples of the bridge form.
2. Cutting the specimen from the supports.
3. Scanning the sample with a laser scanner.
4. Using a scanner program for the geometry comparison analogue of the Gomagic program to determine the field of residual deformations (displacements) in printed and cut samples.
5. Solving the inverse problem of elasticity to determine stresses during known displacements by the FEM.

### 2.2. Printing Samples

For the experiment on determining the effect of the process parameters on the overall strength of the specimens, eight specimens were printed according to the standard. To carry out the research, test specimens were made according to ISO 527-2:2012 [41] under different combinations of process factors.

The printing material was Polylactide (PLA). Polylactide (PLA) is one of the most widely used materials for FDM technology. It is a biodegradable thermoplastic, produced from renewable raw materials, such as corn starch or sugar cane. Table 1 summarises the properties of PLA. It has a lower thermal deformation temperature than other thermoplastics, as well as a lower glass transition temperature. Polylactide possesses a high degree of crystallinity of poly-L-lactide products, reaching 39% [42]. These properties make PLA easy for 3D printing.

The PLA plastic is the second most popular filament, which has a number of undeniable advantages: environmental friendliness and absence of unpleasant odours during printing. In addition, this plastic practically does not shrink. But, the results of printing will be qualitative only when you choose the right parameters and know some nuances. PLA is a rigid plastic which is heavier than ABS is, but it is more brittle when bent.

The task of the study is to determine the influence of printing parameters on the mechanical properties of finished products, as well as to identify the dependence of the level of the residual stresses on the technological factors. For this purpose, a matrix of the three-factor experiment was prepared. The study was carried out on different printing objects, varying the technological parameters one after another. The non-variable factors are summarised in Table 2.

The following factors were taken as varying: the extruder head temperature, filling percentage, and layer thickness. Table 3 and Table 4 show the planning matrix of the experiment.

According to the above standard, the specimens can be of three types: dog-bone-shaped, scapula, and rectangular specimens. It is noted in [43,44,45,46] that large rounding in section transitions can create difficulties when using FDM. This can lead to structural defects, including sharp image breaks, material gaps, and deposition path changes, resulting in anomalous stress peaks and off-axis stress states, especially in thin specimens. In one study [47], it is noted that the experimental data indicate that the use of ASTM D3039 [48] rectangular specimens with straight edges reduces the likelihood of stress concentration-induced failure and the occurrence of abrupt transition zones. Based on these data, a rectangular specimen was selected.

**Figure 1:**
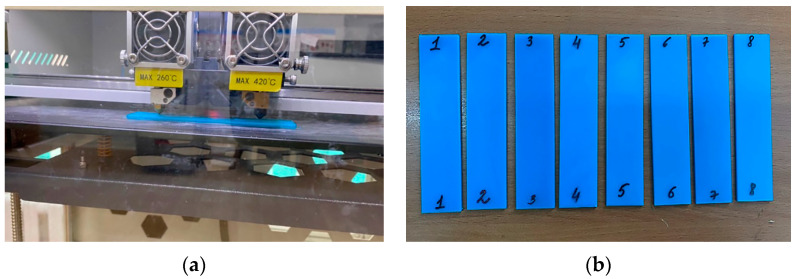
Sample preparation: (**a**) the sample printing process using the CREATE BOT F430 3D printer; (**b**) 8 printed PLA plastic samples.

Sample dimensions are a width of 25 mm, a thickness of 2 mm, a length of 115 mm, and a working length at rupture of 75 mm. The material is PLA plastic. Figure 1 shows photos of the printed samples. The CREATE BOT F430 (Henan Creatbot Technology Limited, Zhengzhou, China) printer was used to print the samples from PLA plastic.

**Figure 2:**
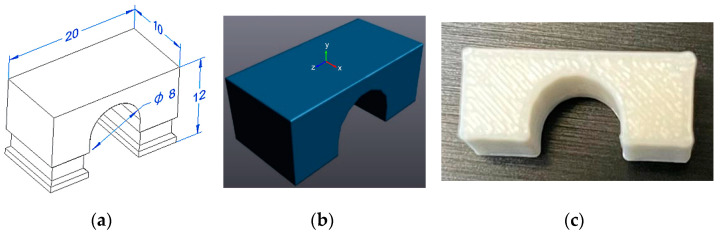
The 3D model of the specimen modelled in the “Solidworks 2018” software. (**a**) Parameters of the bridge model; (**b**) 3D model; (**c**) printed bridge sample.

Eight bridge specimens were printed for the residual stress experiment and the process parameters are summarised in Table 1. Figure 2 shows the 3D model of the standard samples (Figure 2a), the standard samples themselves (Figure 2b), and those printed from PLA plastic (Figure 2c).

Printing was performed with and without the centre support; the model with the support was of better quality.

### 2.3. Scanning of Standard Samples

**Figure 3:**
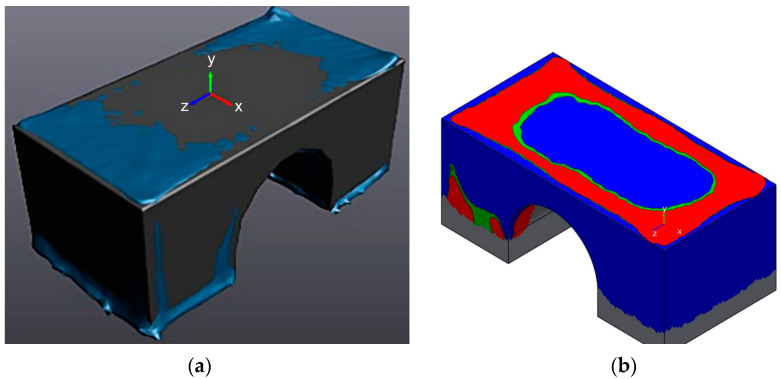

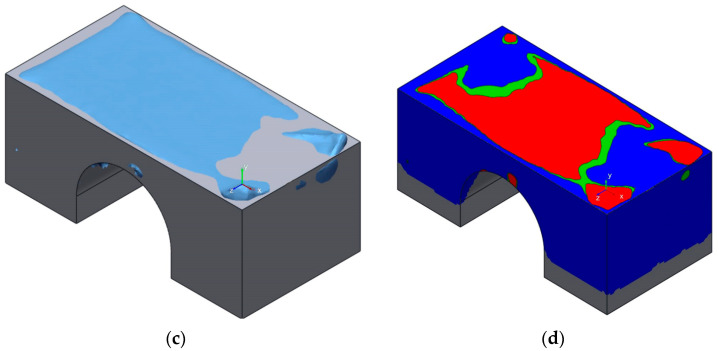
The scanning process using the HandySCAN 3D bridge specimen: (**a**,**b**) scanning process, geometry matching, visualisation of upper fibre compression; (**c**,**d**) geometry matching, visualisation of the tensile strain of the upper fibres.

Scanning was carried out with the HandySCAN 3D scanner (Creaform, Houston, TX, USA). The scanning accuracy in automatic and stationary modes is 0.04 mm, and when using markers in the manual scanning mode, it is 0.05 mm + 0.3 mm/m. Figure 3 shows the superimposed models—the 3D model (grey) and a scan of the printed sample model (blue). The superimposition of the 3D model geometry and the scan of the specimen give not only the values of the displacements in coordinates but also the possibility to determine the nature of the stresses. By visualising the overlapping geometry, the stresses can be differentiated into tensile fibres (red) and compressive fibres (compressed). Fibres in a neutral state are shown in green.

To compare the 3D model with the printed model, the HandySCAN 3D interface is used. The motion estimation of the samples starts with importing the 3D scanner and CAD data using the Best Fit Alignment function. This tool allows you to accurately align 3D scans of objects with the corresponding CAD models for later comparison.

Any deviations outside the set tolerances will be highlighted on the chromatogram, making it easy to identify the area requiring further inspection. The darker the colour, the greater the discrepancy between the 3D scan data and the CAD file. The displacements due to the residual stresses are summarised in Table 5.

It is necessary to note that the order of magnitude of the displacements agrees well with the results provided in [49]. The work contains diagrams of the dependence of the displacements on the printing time; the values of the displacements vary from 0.1 mm to 1.1 mm. But, the author did not provide printing parameters, such as the filling and the layer thickness.

### 2.4. Solution of the Inverse Problem on the Determination of Stresses during Known Displacements by FEM

There are two definitions of the problems of elasticity theories: direct and inverse.

The inverse problem of the theory of elasticity consists of finding the appropriate boundary conditions which correspond to the given continuous functions of the body coordinates, which can be either displacements $u_{i}(x_{k})$ or components of the stress tensor $\sigma_{i}(x_{k})$, satisfying the basic Equations (1)–(3).

The solution of the inverse problem is much simpler than the solution of the direct problem. The inverse problem is especially easy to solve if we specify the displacements $u_{i}(x_{k})$. For given continuous functions $u_{i}\left(x_{k}\right)$, the Saint-Venant joint equations are satisfied.

The inverse problem is solved as follows:

‒ On the basis of geometrical equations, i.e., Cauchy relations (1), the components of the strain tensor are determined:
  (1) $$\varepsilon_{ij}=\frac{1}{2}\left(\frac{\partial u_{i}}{\partial x_{j}}+\frac{\partial u_{j}}{\partial x_{i}}\right);$$
‒ Based on Hooke’s law (2), the components of the stress tensor are determined $\sigma_{ij}(x_{k})$, corresponding to the adopted functions $u_{i}(x_{k})$
  (2) $$\sigma_{ij}=\lambda \delta_{ij}\varepsilon_{kk}+2\mu \varepsilon_{ij},$$
  where $\varepsilon_{kk}=\varepsilon_{11}+\varepsilon_{22}+\varepsilon_{33}$, λ and μ are Lamé coefficients, and $\delta_{ij}$ is the Kronecker symbol;
‒ Based on the equations of equilibrium (1) and boundary conditions, the external forces *f_i_*, when the given displacements are realised, are determined:
  (3) $$\begin{array}{c} \nabla_{j}\sigma_{ij}+\rho f_{i}=\rho {\ddot{u}}_{i};\\ \nabla_{j}\sigma_{ij}+\rho f_{i}=0, \end{array}$$
  where ρ is density, ${\ddot{u}}_{i}$ is the second derivative of the time displacement, and $f_{i}$ is the components of mass forces.

**Figure 4:**
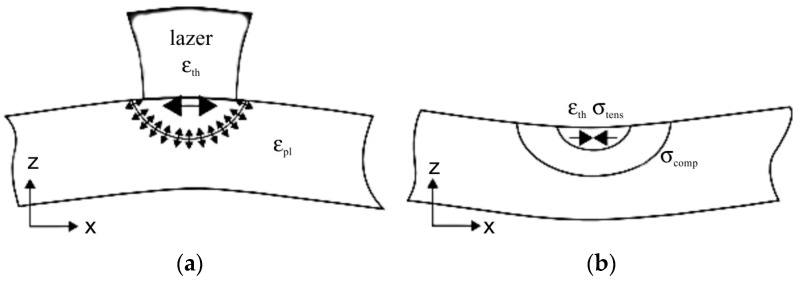
Distribution diagrams of stress components: (**a**) tensile; (**b**) compressive.

In addition to the value of residual stresses, it is also important to know their nature, that is, to find out which components of the stress tensor are tensile or compressive. Figure 4 shows the scheme of the stress state of the plate.

Tensile residual stresses, especially at two- and three-axis stress states, are mostly harmful, while compressive stresses are mostly beneficial.

To estimate the residual stresses in the model, the inverse problem of the elasticity theory was solved in the NASTRAN v11.0 environment. This is a finite element analysis program [50]. The modelling algorithm consisted of the following sequence [51]:

(1) Importing a model;
(2) Finite element approximation;
(3) Setting the boundary conditions:
  ‒ Displacements in the *x*-axis and *y*-axis, according to Table 3;
  ‒ The task of fixing the strategy for creating supports according to the scheme.

The boundary conditions were selected using the coordinates of the strain ellipse (Figure 4), which was determined by comparing the accuracy of the specimen geometry.

## 3. Results

### 3.1. Mechanical Testing of Printed Samples

**Figure 5:**
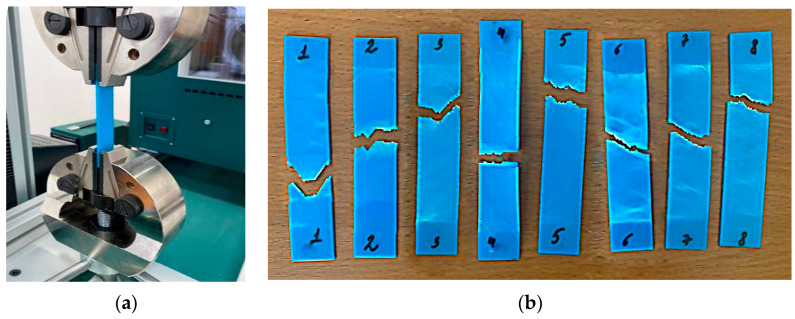
The tensile testing of the samples printed from PLA plastic: (**a**) the experimental setup; (**b**) samples after testing.

**Figure 6:**
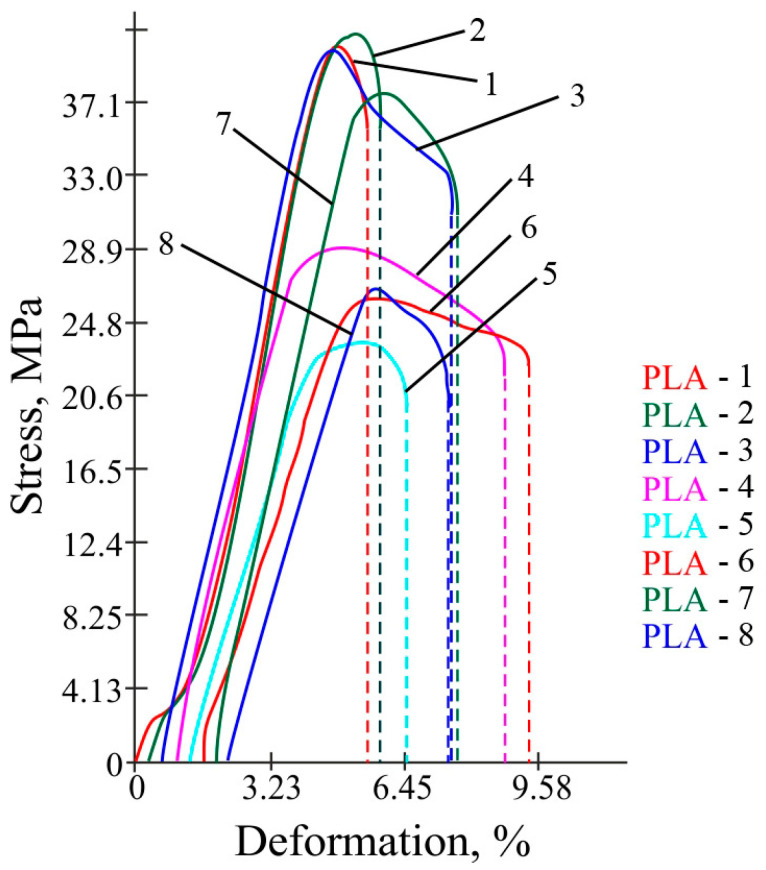
The dependence of stress on deformation for the samples made of PLA plastic at rupture. The curve numbers correspond to the sample numbers from Table 6.

Photos of the experiment and broken samples are shown in Figure 5. Graphs of the stress–strain at rupture are presented in Figure 6.

Sample dimensions are a width of 25 mm, a thickness of 2 mm, a length of 115 mm, and a working length at rupture of 75 mm. The rip speed was 2.00 mm/min; the material was PLA plastic.

The tensile fracture pattern of the specimens shows that the specimens do not always fracture strictly in the centre. This is mainly due to defects in the specimen structure and the deformation behaviour of the test material. Such results are reliable from the point of view of property measurement. An additional factor confirming the reliability of the results is their correlation with the results of work [52].

Data on the results of tensile stresses are summarised in Table 6.

According to the results given in the table, it is obvious that the first three samples have the highest stress at rupture. These samples have an infill density of 100%. The second sample has the highest value of 41.0 MPa. The first and third samples have almost the same stress. In this table, the lowest stress value is for sample number 5 and is equal to 23.6 MPa. The fifth sample has an infill density of 30%. It can be concluded that the infill density has a greater effect on stress. However, the combination of the factors of a 30% fill factor of 0.1 mm layer thickness and temperature of 220 °C gives a stress jump of 37.7 MPa. This fact needs to be investigated in more detail in two-dimensional plots. In order to describe the mathematical model of the dependence of the strength of the printed products on a set of technological parameters, based on the obtained tensile test data, the mathematical processing of the experiment was carried out. The regression Equation (4) was derived and response surfaces were constructed for the dependence of the strength of the polymer samples on the temperature of the extruder head, layer thickness, and filling. The matrix of the three-factor experiment is shown in Table 6.

The equation coefficients were determined (Table 7). Checking the resulting equation for adequacy using the Fisher criterion gives the coefficient of determination equal to R^2^ = 0.95.

Regression equation:

(4) $$y=b_{0}+b_{1}x_{1}+b_{2}x_{2}+b_{3}x_{3}+b_{12}x_{1}x_{2}+b_{13}x_{1}x_{3}+b_{23}x_{2}x_{3}+b_{123}x_{1}x_{2}x_{3}.$$

**Figure 7:**
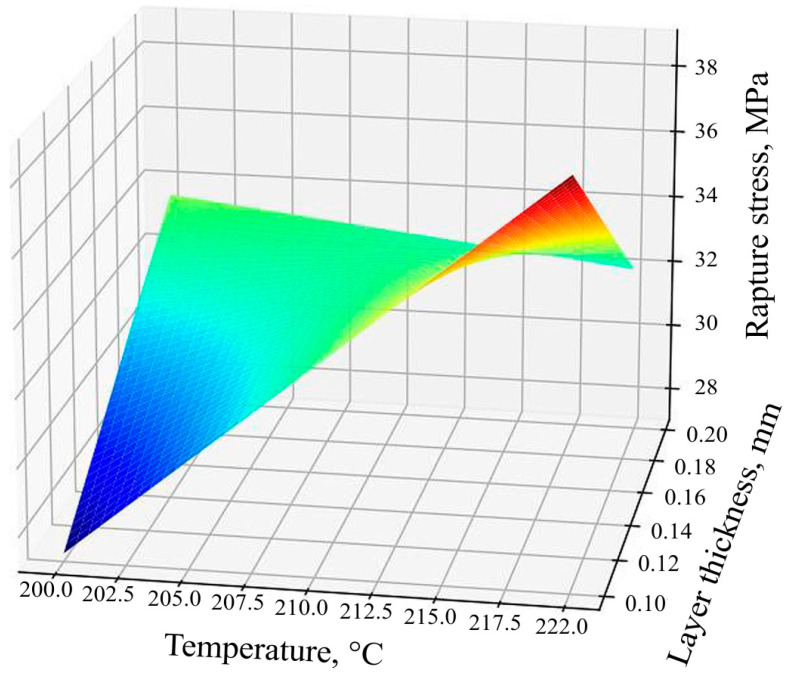
Response surfaces of the tensile strength function of specimens.

Using Equation (4), the response surface (Figure 7) of the dependence of the ultimate tensile strength of the samples based on technological factors was constructed.

The equation shows that the factor *x*_3_ (fill rate) has the strongest influence since it has the largest coefficient in absolute value. After it, in terms of the strength of the influence on the response (tension at break), there is the double interaction of two factors *x*_2 × 3_ (a combination of the factors of the layer thickness and filling) and the least influential factor *x*_1_ (the temperature of the extruder head). Since the coefficients for *x*_1_ and *x*_3_ are positive, then with an increase in these factors, the response increases; i.e., the tensile strength increases. The coefficients for the *x*_2_-layer thickness, *x*_1_*x*_2_, are negative; this means that with a decrease in the *x*_2_-layer thickness factor and the listed interactions, the response value will increase, and with an increase, it will decrease.

### 3.2. Determination of PLA Flexural Strength

**Figure 8:**
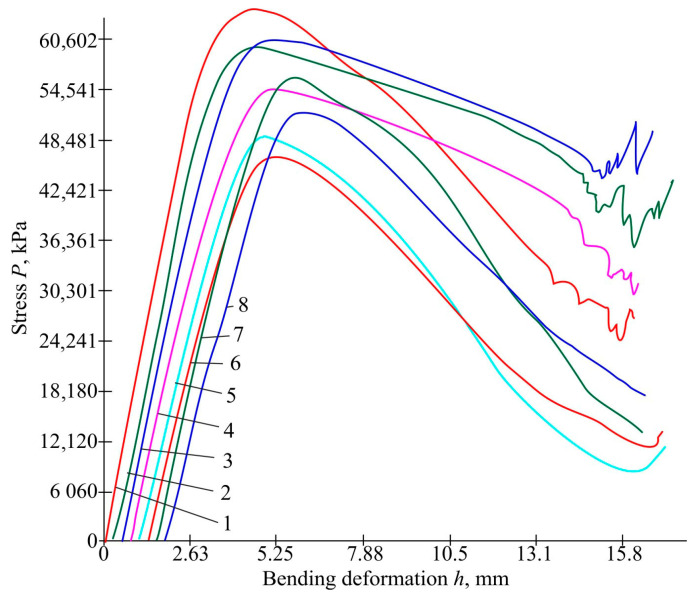
The dependence of stress on displacement for PLA plastic samples during bending (first red stripe—sample No. 1, first green stripe—sample No. 2, first blue stripe—sample No. 3, pink stripe—sample No. 4, blue stripe—sample No. 5, the second red stripe—sample No. 6, the second green stripe—sample No. 7, the second blue stripe—sample No. 8).

**Figure 9:**
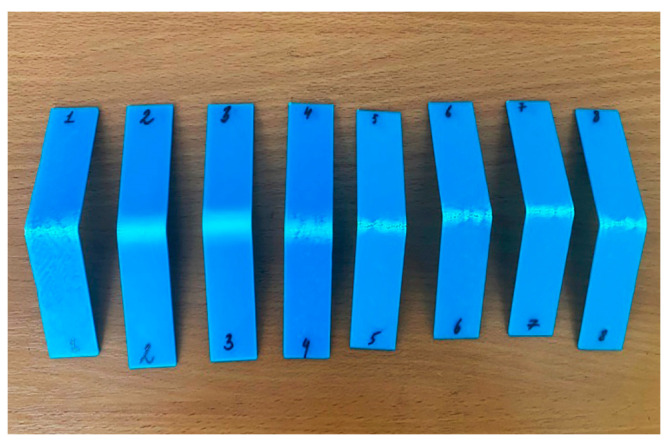
Photos of the specimen bending after mechanical testing.

Graphs for the stress-displacement during bending for the PLA samples are presented in Figure 8. Photos of specimen bending before and after are shown in Figure 9.

The data on the results are summarised in Table 8.

Table 6 shows that with almost identical displacements of the plate sections, the highest stress value is for the sample number one and it is equal to 64.542 MPa. For the first sample, all experimental parameters have the upper value. The stress of the second and third samples is almost the same and the stress values are slightly lower than those of the first sample. These two have 100% infill density. The fourth, seventh, and eighth samples have an average voltage value. And the lowest values are for the fifth and sixth samples, 48.985 and 46.603 MPa, respectively. The infill density of these samples is 30%. The conclusion is that the infill density affects the bending stress.

Based on the obtained data, Table 9 was compiled and shows the planning matrices for processing the results of the PLA plastic.

The mathematical model of the dependence of bending strength on technological parameters is described by Equation (5). Determining the coefficients and checking the resulting equation for adequacy using the Fisher criterion give an understanding of the adequacy of the mathematical model. The equation coefficients were determined (Table 10).

The regression equation is:

(5) $$y=b_{0}+b_{1}x_{1}+b_{2}x_{2}+b_{3}x_{3}+b_{12}x_{1}x_{2}+b_{13}x_{1}x_{3}+b_{23}x_{2}x_{3}+b_{123}x_{1}x_{2}x_{3}.$$

**Figure 10:**
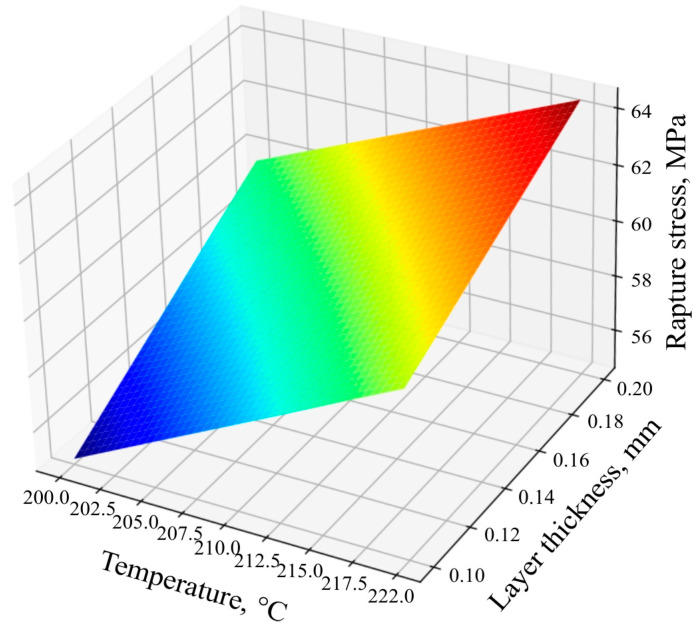
The response surface of the flexural strength dependence of specimens.

Using Equation (5), the response surface (Figure 10) of the ultimate bending strength dependence of samples on technological factors was constructed

The equation shows that the factor *x*_3_ (filling rate) has the strongest influence since it has the largest coefficient in absolute value. After it, in terms of the strength of influence on the response (tension at break), there are factors *x*_2_*x*_3_ (a combination of the factors of the layer thickness and filling rate). Further, according to the weight of the contribution, factor *x*_1_ is the temperature of the extruder head. Since the coefficients for *x*_1_ and *x*_3_ are positive, then with an increase in these factors, the response increases; i.e., the bending strength increases. The coefficients for *x*_2_, *x*_1_*x*_2_ are negative, which means that with a decrease in the *x*_2_ factor, that is, the thickness of the layer and the listed interactions, the response value will increase, and it will decrease with an increase.

**Figure 11:**
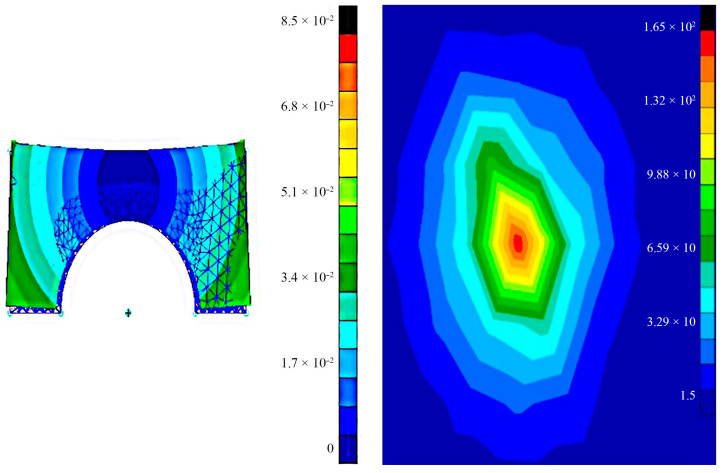
The diagram of stresses and displacements of a bridge printed using a combination of different technological factors.

Figure 11 shows graphs of the dependence of the bending strength on the technological factors infill density, layer thickness, and extruder head temperature.

The complete filling of the part with material allows you to increase the bending strength 1.5 times; however, the mass of the product will increase 2.5 times compared to a filling of 30%.

## 4. Discussion

Based on the results of solving the inverse problem of the theory of elasticity, the stresses causing displacement of the sections of the bridge structure, printed with a combination of various technological factors given in the table, were determined. Figure 11 shows a diagram of the stress–strain state of the bridge.

**Figure 12:**
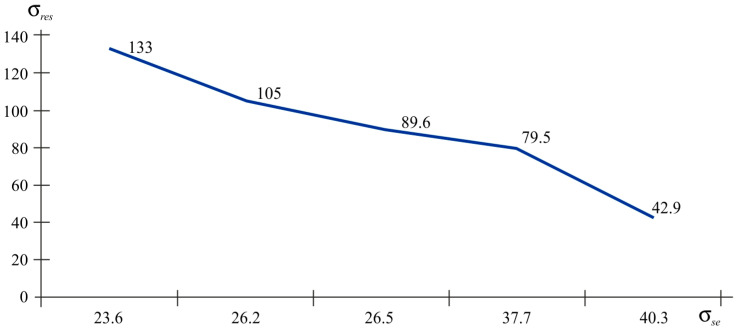
The residual stress–fibre tensile strength stress graph.

To process the results of determining the residual stresses, deformations and stress values were differentiated by nature into tensile and compressive. Table 9 shows the values of tensile stresses; Table 11 and Table 12 summarises the results of compressive and strength stresses. Figure 12 demonstrate the strength of the samples as a function of tensile and compressive residual stresses. The index *res* shows the residual stresses and the index *st* provides the stresses of the experimental tensile strength.

According to the graphs presented in Figure 12, it is obvious that with the tensile nature of the residual stresses, the relationship between residual stress and strength stress is inversely proportional. If we talk about technological factors, then samples 5, 6, 7, and 8 are united by one factor (packaging or infill density). That is, with a filling density of 30%, tensile stresses arise in printed products. Despite the fact that the residual stress limit exceeds the tensile strength, there are no visible defects. These micro-residual stresses reach peak values exceeding the plastic yield strengths of 42 and 64 MPa but without damage. The maximum tensile stress value corresponds to the sample with 100% packing; the residual tensile stress reaches a minimum value of 41.9 MPa. These micro-residual stresses reduce the strength by at least 16%, while the elastic properties remain virtually unchanged.

These results are in good agreement with the results of M.P. Danilaev, S.A. Karandashov, A.G. Kiyamov et al. [52]. They note in their studies that the results of calculating normal radial and tangential residual stresses show that with a slight change in the degree of orthotropy in the range of k_orth_ = 0.95–1.05, the values of the tangential $\sigma_{\theta \theta }$ and radial $\sigma_{rr}$ residual stresses can reach a significant value (0.15 ÷ 0.20)E_r_.

**Figure 13:**
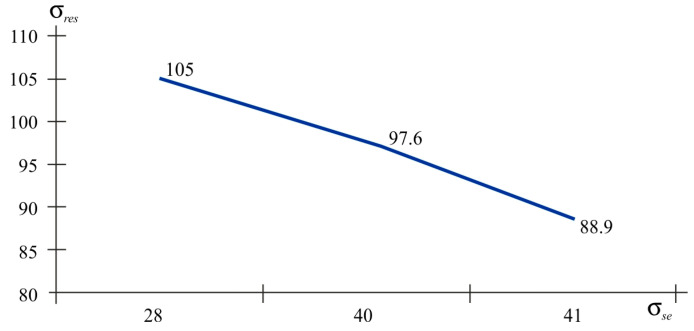
The graph of the relationship between residual stress and compressive strength stress of fibres.

Also in [14], the results of computer modelling of the residual stresses arising during the technological process of printing from ABS plastic are presented. The authors used the Digimat-AM program; computer analysis showed a residual stress level of 76 MPa. Figure 13 shows data on residual stress and stresses at rupture for different technological factors, which are given in the experiment matrix. The difference between the results of the computational experiment and the physical experiment is justified in [3]. Based on a comparison of the tensile strength data in the Digimat-ME program and the results of the tests, the technology was found to provide a 20% reduction in strength indicators.

Analysis of the graphs in Figure 13 reveals the inverse relationship between the values of residual stresses and strength stresses. All samples have a 100% filling rate and the sample with the highest strength index has a layer thickness of 0.1 mm.

When comparing the values of the same strength stresses in the graphs (Figure 12 and Figure 13), it is obvious that with similar strength values, the tensile nature of the stresses has a residual stress value of 42.9 MPa. This is lower than it is when the fibres are compressed at a value of 88.9 MPa. That is, it can be argued that compressive stresses have a positive effect on tensile strength.

The mathematical analysis of the results of the experiment on determining the level of the residual stresses based on the technological parameters of the printing process made it possible to determine the functional dependence of the residual stresses. A set of parameters in Table 13 shows the results obtained during the simulation.

**Figure 14:**
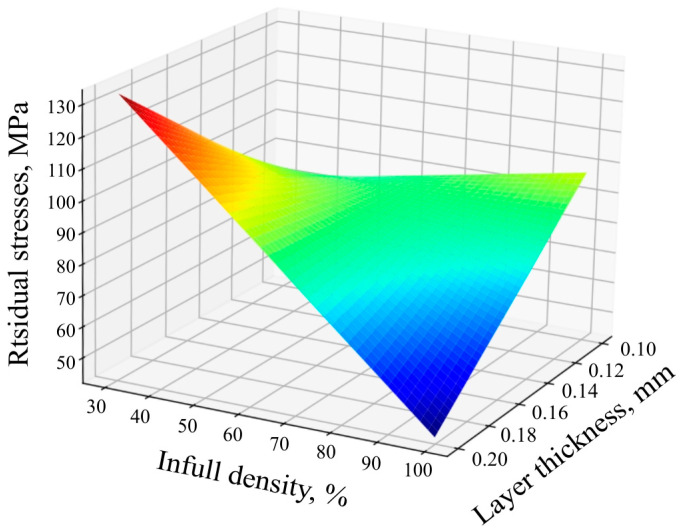
Response surfaces of the residual stress function.

The results of regression analysis using Formula (6) shows that the infill density greatly affects the residual stress, while according to [14], the filling structure does not affect the residual stress. The equation coefficients were determined (Table 14). Figure 14 shows the response surface of the dependence of the residual stresses on the printing technological factors:

(6) $$y=b_{0}+b_{1}x_{1}+b_{2}x_{2}+b_{3}x_{3}+b_{12}x_{1}x_{2}+b_{13}x_{1}x_{3}+b_{23}x_{2}x_{3}+b_{123}x_{1}x_{2}x_{3}.$$

**Figure 15:**
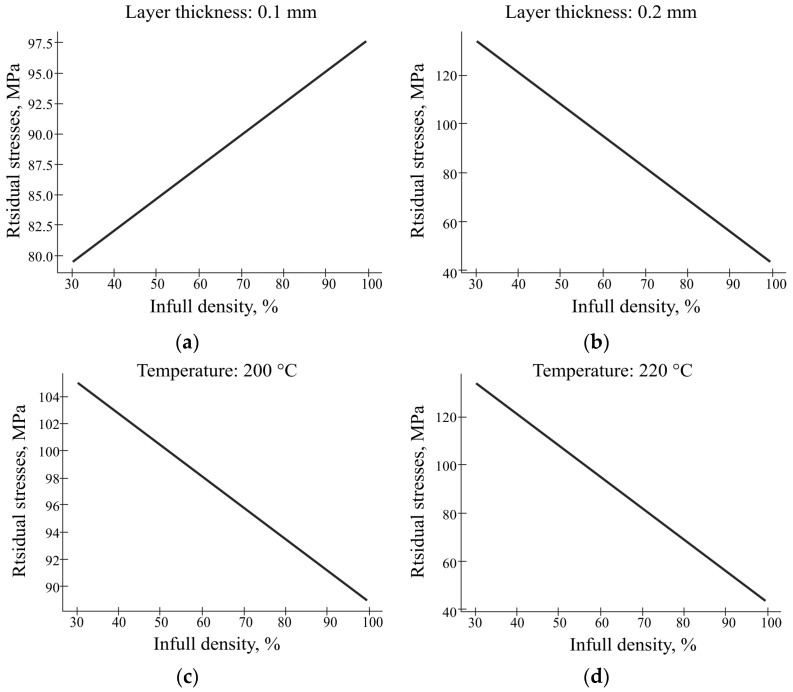
The graphs of residual stresses versus infill density: (**a**) at a fixed temperature of 200 °C; (**b**) at a fixed temperature of 220 °C; (**c**) with a layer thickness of 1 mm; (**d**) with a layer thickness of 2 mm.

The graph in Figure 15 illustrates the relationship between the residual stress and design parameters. When the layer thickness is 1 mm, the magnitude of the residual stress increased due to the increase in infill density, which was due to the increase in heat generation and temperature that changed the design. With a layer thickness of 0.2 mm, we observe an inversely proportional dependence of the residual stresses on the filling rate. The results of this study showed that the level of infill density significantly influences the residual stress in FDM-printed PLA parts. With a low infill density of 30% and a layer thickness of 1 mm, the residual stress was measured to be approximately 97.5 MPa and had a lower value while at a higher infill density of 100% and a layer thickness of 2 mm; the residual stress increased significantly and was approximately 110 MPa. In other words, it cannot be said that the density unambiguously inversely affects the level of the residual stresses, as was emphasised in previous works. Additional factors such as layer thickness must be taken into account. An inverse proportional relationship is observed in the combination of the layer thickness of 2 mm and temperature of 200 °C.

**Figure 16:**
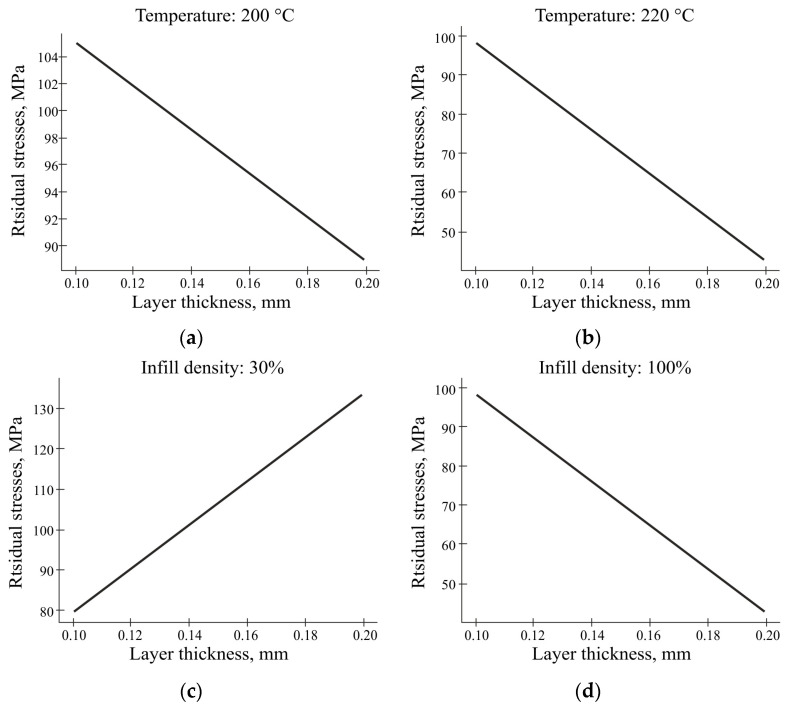
The graphs of the residual stresses versus the layer thickness: (**a**) at a fixed temperature of 200 °C; (**b**) at a fixed temperature of 220 °C; (**c**) at a fixed filling of 30%; (**d**) at a fixed filling of 100%.

In this study, the relationship between the layer thickness and residual stress was investigated, and the results were consistent with previous results. As shown in Figure 16a,b, it is obvious that the layer thickness has an inverse relationship with the residual stress under the combined influence of temperature factors and the layer thickness. When studying the combined influence of the filling factors and the layer thickness, the opposite picture is observed (Figure 16c,d) in the case of 30% filling. That is, with this filling option, an increase in the layer thickness leads to an increase in the residual stresses. This means that with an increase in the layer thickness, the residual stress decreases. This relationship was also observed in another study [50]. A high level of stress can negatively affect the performance characteristics of the printed parts; even if it does not lead to visible damage, it will affect the geometry of the printed parts. These results suggest that optimizing the layer thickness can be an effective way to reduce the residual stresses and improve the mechanical properties of FDM-printed parts.

**Figure 17:**
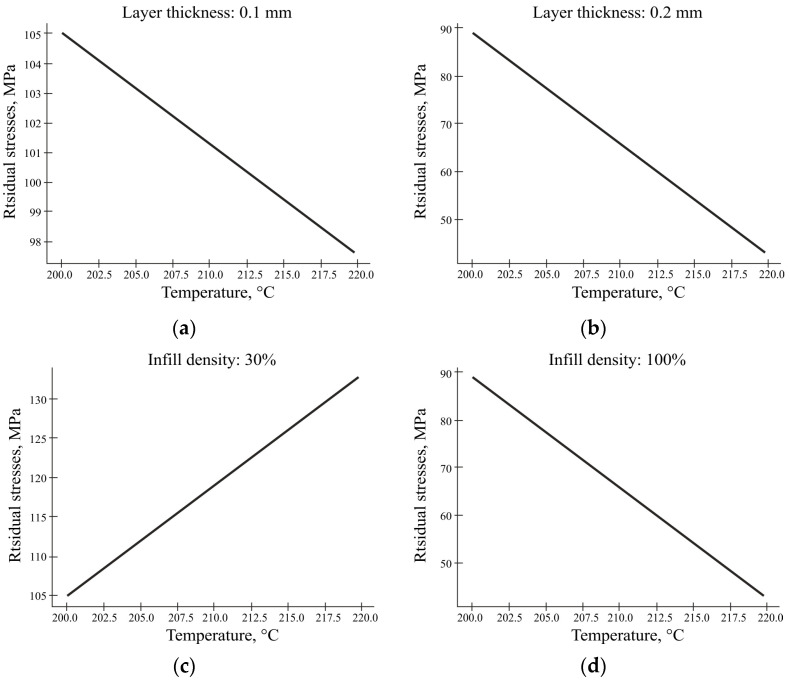
The graph of residual stress versus temperature: (**a**) with a fixed layer thickness of 0.1 mm; (**b**) with a fixed layer thickness of 0.2 mm; (**c**) with a fixed filling of 30%; (**d**) with a fixed filling of 100% according to the schedules.

The printing temperature increases the residual stress if the extrusion process occurs at the lowest fixed density of 30% (Figure 17c). The increase in temperature at low density causes stronger expansion before solidification and then faster contraction when the filling cools, which introduces changes in the crystallisation process. And at the higher density of 100% (Figure 17d), the cooling of the layers is slower. These findings are in agreement with those of [15] that heat transfer between tracks and application time between layers are crucial to obtain low-strain parts. The inverse relationship between the residual stresses and tempirature is observed in the combination of a fixed factor with layer thickness (Figure 17a,b).

Taking into account all the derived relationships between the magnitude of the residual stresses, their nature, and technological factors, a removable matrix for bending sheet metal was designed and tested. The most favourable combination of factors for reducing residual stresses is the occupancy rate of 100% according to the graphs in Figure 15, the layer thickness of 0.2 mm according to Figure 16, and the temperature of 220 °C.

If we talk about the nature of the residual stresses, then the forming surfaces of the matrix will experience tensile deformation of the upper fibres owing to the bending force. It is assumed that they will not be mitigated by the residual compressive stresses that arise with such combination of technological factors.

## 5. Conclusions

Based on the conducted research, the following conclusions were made:

- The proposed combined method for determining residual stresses can be used to study residual stresses in polymer parts.
- Experimental studies and practical experience show that compressive residual stresses increase the strength in parts printed from PLA plastic, while tensile residual stresses have an adverse effect.
- The effect of the residual stresses on strength depends on the mechanical properties of the material and the nature of the stress state. With significant compressive stresses in the surface layer, an increase in strength is observed.
- Contrary to expectations, there is no monotonic increase in strength with increasing infill density of the printed parts. The observed decrease in strength when the filling is close to 50% requires special consideration.
- By creating controlled residual stresses, which are subtracted from operating stresses, the performance properties of the material can be improved. Most often, compressive residual stresses are deliberately created in the surface layer, which reduces dangerous tensile operating stresses.
- The obtained results are applicable to the design of the FDM-printed polymer injection tool.

The resulting dependence of the residual stress on the process factors can help manufacturers optimize print quality, minimize part warping, and achieve the desired mechanical properties of the printed parts.

## Figures and Tables

**Table 1:** PLA properties.

Name	Specification
Material	PLA
Wire diameter	1.75 ± 0.05 mm
Density	800 g
Melting point	170–180 °C
Glass transition temperature	600–650

**Table 2:** The non-variable parameters of printing.

Parameters	
Nozzle diameter	0.4 mm
Printed raster	–45°/45°
Max. print speed	50 mm/s
Platform temperature	60 °C

**Table 3:** Parameters of the experiment of the layer-by-layer PLA deposition.

Parameters	Max	Min
Temperature, *x*_1_, °C	220	200
Thickness of layer, *x*_2_, mm	0.2	0.1
Filling percentage, *x*_3_, %	100	30

**Table 4:** Parameters of the 3-factor PLA experiment.

No.	Temperature, °C	Thickness of Layer, mm	Infill Density, %	*x*_1_	*x*_2_	*x*_3_
1	+	+	+	220	0.2	100
2	−	+	+	200	0.2	100
3	+	−	+	220	0.1	100
4	−	−	+	200	0.1	100
5	+	+	−	220	0.2	30
6	−	+	−	200	0.2	30
7	+	−	−	220	0.1	30
8	−	−	−	200	0.1	30

**Table 5:** Scanning data for the movement of bridge sections.

No. of Points	*x*-Axis	No. of Points	*y*-Axis
1	0.148	1	0.167
2	−0.062	2	0.047
3	−0.007	3	0.041
4	0.052	4	0.076
5	0.074	5	0.065
6	0.071	6	0.059
7	0.063	7	0.067
8	0.047	8	0.077
9	0.039	9	0.073
10	−0.003	10	0.020
11	−0.026	11	0.019
12	−0.082		
13	0.227		

**Table 6:** Tensile stress along PLA layers.

No.	Temperature, °C	Layer Thickness, mm	Infill Density, %	*x*_1_	*x*_2_	*x*_3_	σ_v_ Tensile Strength, MPa	Y Young’s Modulus, MPa
1	+	+	+	220	0.2	100	40.3	1130
2	−	+	+	200	0.2	100	41.0	1100
3	+	−	+	220	0.1	100	40.1	1090
4	−	−	+	200	0.1	100	28.0	1370
5	+	+	−	220	0.2	30	23.6	1000
6	−	+	−	200	0.2	30	26.2	1050
7	+	−	−	220	0.1	30	37.7	1090
8	−	−	−	200	0.1	30	26.5	1090

**Table 7:** Regression equation coefficient values for calculating strength.

$b_{0}$	$b_{1}$	$b_{2}$	$b_{3}$	$b_{12}$	$b_{13}$	$b_{23}$	$b_{123}$
32.925	2.500	−0.150	4.425	−3.325	0.350	3.450	0.125

**Table 8:** Bending stress of PLA plastic samples.

No. Sample	Factors of Influence			Maximum Stress, MPa
	Temperature, °C	Layer Thickness, mm	Infill Density, %	
1	220	0.2	100	64.5
2	200	0.2	100	59.6
3	220	0.1	100	60.6
4	200	0.1	100	54.7
5	220	0.2	30	48.9
6	200	0.2	30	46.6
7	220	0.1	30	56.0
8	200	0.1	30	51.9

**Table 9:** The matrix for processing the results of the bending experiment on plastic samples.

No.	Temperature, °C	Layer Thickness, mm	Infill Density, %	*x*_1_	*x*_2_	*x*_3_	Maximum Stress, MPa
1	+	+	+	220	0.2	100	64.542
2	−	+	+	200	0.2	100	59.654
3	+	−	+	220	0.1	100	60.601
4	−	−	+	200	0.1	100	54.789
5	+	+	−	220	0.2	30	48.985
6	−	+	−	200	0.2	30	46.603
7	+	−	−	220	0.1	30	56.013
8	−	−	−	200	0.1	30	51.947

**Table 10:** Regression equation coefficient values for calculating bending strength.

$b_{0}$	$b_{1}$	$b_{2}$	$b_{3}$	$b_{12}$	$b_{13}$	$b_{23}$	$b_{123}$
55.392	2.144	−0.446	4.505	−0.326	0.532	2.647	0.095

**Table 11:** The value of tensile residual stresses and tensile strength stresses.

No. Sample (Combination of Factors)	$\sigma_{res}$, MPa	$\sigma_{st}$, MPa
1 (220/0.2/100)	42.9	40.3
5 (220/0.2/30)	133	23.6
6 (200/0.2/30)	105	26.2
7 (220/0.1/30)	79.5	37.7
8 (200/0.1/30)	89.6	26.5

**Table 12:** The value of compressive residual stresses and tensile strength stresses.

No. Sample	$\sigma_{res}$	$\sigma_{st}$
2 (200/02/100)	88.9	41.0
3 (220/01/100)	97.6	40.1
4(200/01/100)	105	28.0

**Table 13:** The matrix for processing experimental results to determine residual stresses in plastic samples.

No.	Temperature, °C	Layer Thickness, mm	Infill Density, %	*x*_1_	*x*_2_	*x*_3_	Y Tension at Break, MPa	Residual Stress, MPa
1	+	+	+	220	0.2	100	40.3	42.9
2	−	+	+	200	0.2	100	41.0	88.9
3	+	−	+	220	0.1	100	40.1	97.6
4	−	−	+	200	0.1	100	28.0	105
5	+	+	−	220	0.2	30	23.6	133
6	−	+	−	200	0.2	30	26.2	105
7	+	−	−	220	0.1	30	37.7	79.5
8	−	−	−	200	0.1	30	26.5	89.6

**Table 14:** Values of the coefficients of the regression equation of the response surface of the dependence of residual stresses on printing technological factors.

$b_{0}$	$b_{1}$	$b_{2}$	$b_{3}$	$b_{12}$	$b_{13}$	$b_{23}$	$b_{123}$
92.6875	−4.438	−0.237	−9.088	−0.063	−8.913	−17.463	−9.587

## Data Availability

The data presented in this study are available from the corresponding authors upon reasonable request.
